# Study of Resting-State Functional Connectivity Networks Using EEG Electrodes Position As Seed

**DOI:** 10.3389/fnins.2018.00235

**Published:** 2018-04-24

**Authors:** Gonzalo M. Rojas, Carolina Alvarez, Carlos E. Montoya, María de la Iglesia-Vayá, Jaime E. Cisternas, Marcelo Gálvez

**Affiliations:** ^1^Laboratory for Advanced Medical Image Processing, Department of Radiology, Clínica las Condes, Santiago, Chile; ^2^Medical Bio-Modeling Laboratory, Department of Radiology, Clínica las Condes, Santiago, Chile; ^3^Department of Radiology, Clínica las Condes, Santiago, Chile; ^4^Advanced Epilepsy Center, Clínica las Condes, Santiago, Chile; ^5^Department of Paediatric Neurology, Clínica las Condes, Santiago, Chile; ^6^Joint Unit FISABIO & Prince Felipe Research Center (CIPF), Valencia, Spain; ^7^Centro de Investigación Biomédica en Red de Salud Mental (CIBERSAM-G23), Madrid, Spain; ^8^Hospital of Sagunto, Valencia, Spain; ^9^School of Engineering and Applied Sciences, Universidad de los Andes, Santiago, Chile

**Keywords:** functional connectivity, EEG, rs-fMRI, EEG-fMRI, 10-10 EEG system, 10-20 EEG system

## Abstract

Electroencephalography (EEG) is the standard diagnosis method for a wide variety of diseases such as epilepsy, sleep disorders, encephalopathies, and coma, among others. Resting-state functional magnetic resonance (rs-fMRI) is currently a technique used in research in both healthy individuals as well as patients. EEG and fMRI are procedures used to obtain direct and indirect measurements of brain neural activity: EEG measures the electrical activity of the brain using electrodes placed on the scalp, and fMRI detects the changes in blood oxygenation that occur in response to neural activity. EEG has a high temporal resolution and low spatial resolution, while fMRI has high spatial resolution and low temporal resolution. Thus, the combination of EEG with rs-fMRI using different methods could be very useful for research and clinical applications. In this article, we describe and show the results of a new methodology for processing rs-fMRI using seeds positioned according to the 10-10 EEG standard. We analyze the functional connectivity and adjacency matrices obtained using 65 seeds based on 10-10 EEG scheme and 21 seeds based on 10-20 EEG. Connectivity networks are created using each 10-20 EEG seeds and are analyzed by comparisons to the seven networks that have been found in recent studies. The proposed method captures high correlation between contralateral seeds, ipsilateral and contralateral occipital seeds, and some in the frontal lobe.

## Introduction

Electroencephalography (EEG) is a method that measures the electrical activity of the brain. EEG uses surface electrodes to measure the electrical brain signals (Tatum et al., 2008; Schomer and Lopes da Silva, 2011).

EEG is routinely used to diagnose or monitor the following medical conditions and diseases: differentiate epileptic seizures, pre-operative assessment for defining an eventually resectable epileptogenic zone, tumors, differential diagnosis of paroxysmal events, analysis of encephalopathies, sedated patients at risk of seizures, prognosis cardiac arrest and hypoxic ischemic encephalopathy (HIE), brain death diagnosis, psychomotor regression study, acoustic or language development, and sleep disorders (Bickford, 1987). EEG is used for research purposes in the following topics: neuromarketing, psychology (processes underlying attention, learning and memory).

**Figure 1:**
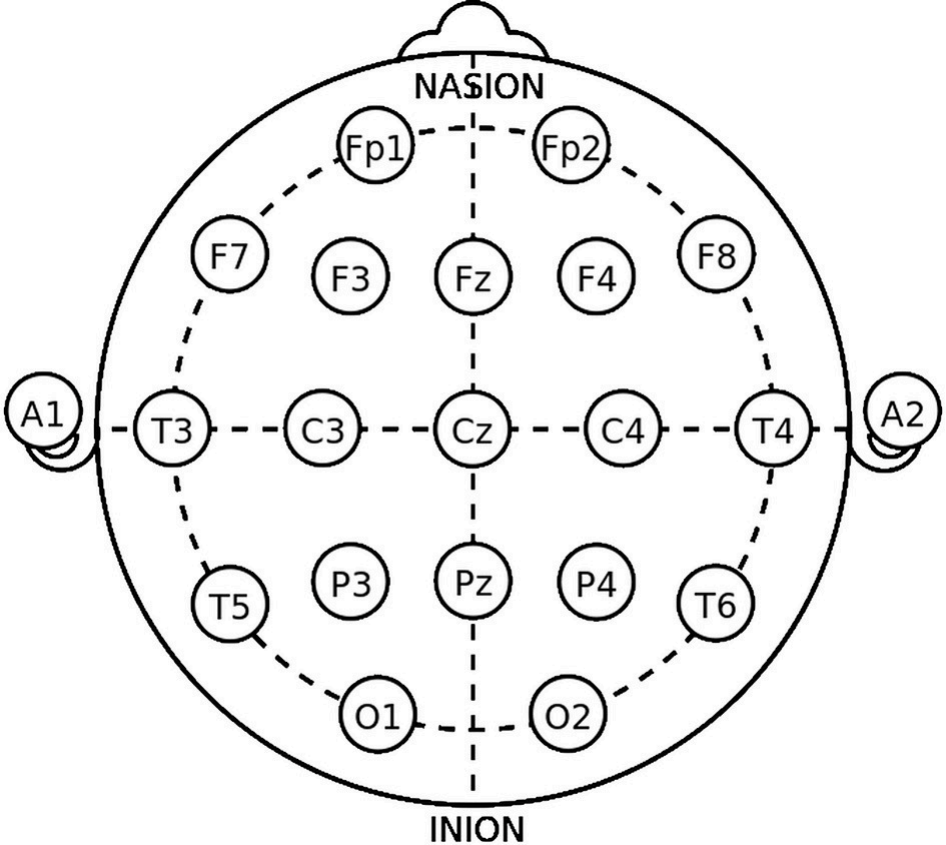
The 10-20 International system of EEG electrode placement.

**Figure 2:**
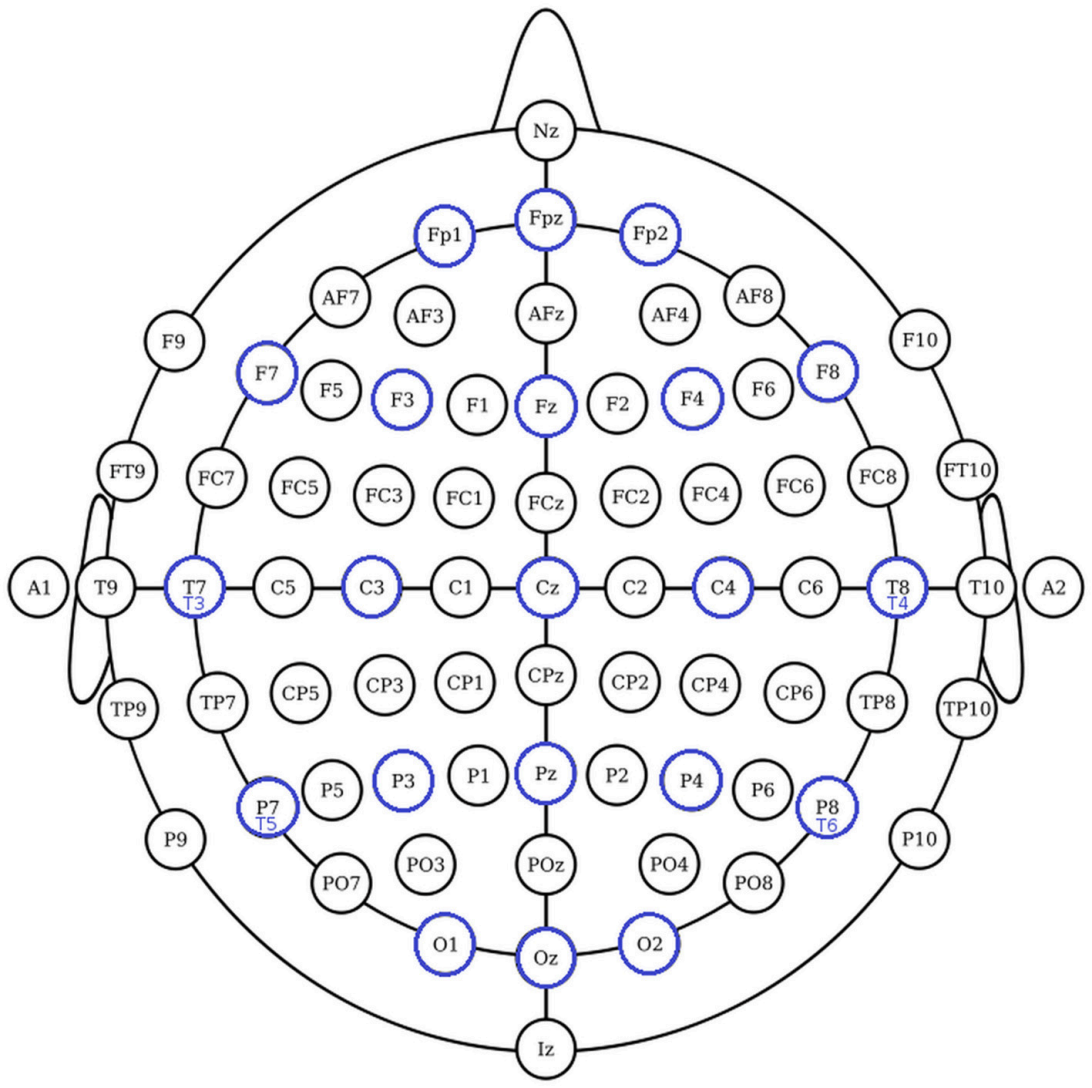
The 10-10 International system of EEG electrode placement. Blue circles represents the location of 10-20 EEG electrodes. Nodes T8, T7, P8, and P7 from 10-10 EEG placement are equivalent to nodes T4, T3, T6, T5 from 10-20 EEG placement.

The International Federation of Clinical Neurophysiology (http://www.ifcn.info/) adopted the standardization for EEG electrode placement called 10–20 electrode placement protocol (Jasper, 1958; Klem et al., 1999). This protocol standardized the physical placements and designations of 21 electrodes on the scalp. Using reference points on the skull in the nasion, preauricular points and inion (Figure 1), the head is divided into proportional positions to provide adequate coverage of all the brain regions. The name of each electrode consists of a letter and a number. The letter refers to the region of the brain where the electrode is positioned (F: frontal, C: central, T: temporal, P: posterior, and O: occipital), and the number is related to the cerebral hemisphere (even numbers in the right hemisphere, and odd numbers in the left; Figure 1). In 1985, an extension to the original 10-20 system was proposed involving an increase in the number of electrodes from 21 to 74 (Figure 2) (Chatrian et al., 1988; American Electroencephalographic Society, 1994; Nuwer et al., 1998; Klem et al., 1999). 10-20 EEG electrode placement system is considered for clinical use, and 10-10 is more used for research.

Functional connectivity MRI (fcMRI) measures the intrinsic functional correlations between brain regions (Van Dijk et al., 2010; Mueller et al., 2013). The method is sensitive to the coupling of both distributed as well as adjacent areas (Biswal et al., 1995; Yeo et al., 2011). It is believed that low-frequency fluctuations observed in the BOLD signals reflect the spontaneous neural activity and that the synchronized fluctuations in distinct brain regions, therefore, point to functional connections between them. Different functional connectivity networks have been found, and these networks change in patients with multiple pathologies (neurological, psychiatric). This renders fcMRI an interesting technique to further our understanding of brain function in health and disease. There are several methods for processing the BOLD signal to obtain connectivity networks: ICA (Independent Component Analysis) (Beckmann et al., 2005; Calhoun and Adali, 2006), seed-based method using correlation between BOLD time series (Biswal et al., 1995; Margulies et al., 2007; Van Dijk et al., 2010), ALFF (Amplitude of Low-Frequency Fluctuations; Zang et al., 2007).

**Figure 3:**
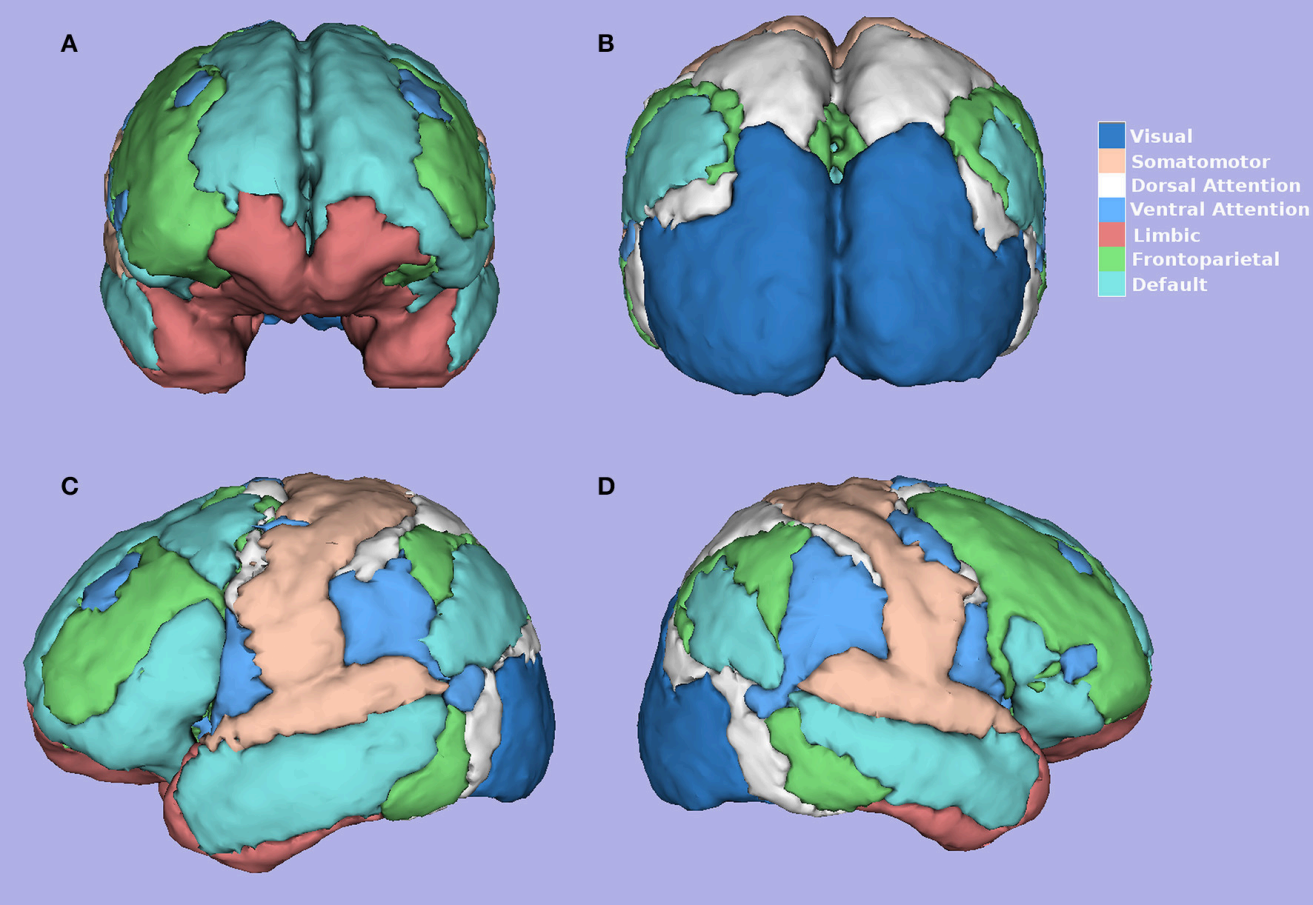
Seven Yeo Networks (Yeo et al., 2011). **(A)** Frontal, **(B)** Posterior, **(C)** Left, **(D)** Right side view. Figure created using software 3D-Slicer.

Yeo et al. (2011), using resting-state data of 1,000 healthy individuals, with 1,175 ROIs on the cortex, the correlation between the fMRI time series of each ROI, and a clustering algorithm demonstrated the existence of seven main functional networks and a finer solution for 17 functional networks. The seven functional networks are visual, somatomotor, dorsal attention, ventral attention, limbic, frontoparietal, and default mode networks (Figure 3; Yeo et al., 2011). The networks found by Yeo et al. (2011) have shown to be valid across multiple subjects and robust to changes in the data processing. The existence of these networks provides a key motivation for our research.

One difference between fMRI and EEG is that fMRI has an excellent spatial resolution and a low temporal resolution (seconds), while EEG has a high temporal resolution (milliseconds) with spatial limitations. Simultaneous acquisition of fMRI and EEG could be a solution for the temporal limitations of fMRI and spatial limitations of EEG by combining their features (Zang et al., 2007; Ullsperger and Debener, 2010). Many authors show different acquisition techniques and data analysis for simultaneous EEG and fMRI (Lazeyras et al., 2001; Menon and Crottaz-Herbette, 2005; Ullsperger and Debener, 2010; Duyn, 2012; Huster et al., 2012; Han et al., 2014). Other difference between both techniques are: EEG measures directly the electrical activity of the brain and fMRI indirectly by measuring changes in blood flow. EEG can be acquired simultaneously with fMRI (high-temporal-resolution data with high-spatial-resolution respectively; Lazeyras et al., 2001; Menon and Crottaz-Herbette, 2005; Ullsperger and Debener, 2010; Duyn, 2012; Han et al., 2014).

The fMRI acquired -either simultaneously or not- with an EEG of the same patient, could be processed in many different ways, for instance, ICA (data-driven; Beckmann et al., 2005; Calhoun and Adali, 2006), ALFF (Zang et al., 2007), and correlation analysis (seed-based method; Biswal et al., 1995; Margulies et al., 2007; Van Dijk et al., 2010).

One of the methods to combine EEG with fMRI is to perform a good signal EEG acquisition within the MRI equipment. But, that type of acquisition inside MRI scanner produces an artifact in EEG signal called “gradient artifacts.”There are different algorithms to remove gradient artifacts from the EEG signal, and statistical algorithms to process the combined EEG-fMRI signal (Gotman et al., 2004, 2006).

In this technical article, we show the functional connectivity networks obtained using seeds relative to the position of 10-20 and 10-10 EEG electrodes, and the relationship of these networks to seven functional connectivity networks (Yeo et al., 2011).

## Materials and methods

### Subjects

We processed rs-fMRI scans of 45 right-handed healthy volunteers (18–27 years, 10 male and 35 female, TR = 3,000 ms, slices = 47, # timepoints = 119, 3T MRI; Cambridge-Buckner dataset, 1,000 Functional Connectomes Project; http://fcon_1000.projects.nitrc.org/), young healthy volunteers used in a previous work.

### fMRI data processing and time series analysis

#### Image preprocessing

Data processing was performed using the Analysis of Functional NeuroImages software (AFNI; http://afni.nimh.nih.gov/afni; Cox, 1996; Cox and Hyde, 1997) and fMRIB Software Library (FSL; http://fsl.fmrib.ox.ac.uk/fsl/fslwiki; Smith et al., 2004; Jenkinson et al., 2012). Image preprocessing involved the following steps: discarding the first 4 EPI volumes from each resting state scan to allow for signal equilibration; slice-time correction for interleaved acquisitions; 3-D motion correction with Fourier interpolation (volumes in which head motions caused a displacement of more than 2 mm in the x, y, or z-direction, or in which 2° of any angular motion was observed during the course of the scan, were excluded); despiking (detection and removal of extreme time series outliers); spatial smoothing using a 6 mm FWHM Gaussian kernel; mean-based intensity normalization of all volumes by the same factor; temporal bandpass filtering (0.009–0.1 Hz); and linear and quadratic detrending.

FSL *FLIRT* was used for linear registration of the high-resolution structural images to the MNI152 template (Jenkinson and Smith, 2001; Jenkinson et al., 2002). This transformation was then refined using *FNIRT* non-linear registration (Andersson et al., 2007a,b). Linear registration of each participant's functional time series to the high-resolution structural image was performed using *FLIRT*. This functional-to-anatomical co-registration was improved by intermediate registration to a low-resolution image and b0 unwarping.

#### Nuisance signal regression

To control the effects of motion and physiological processes (related to cardiac and respiratory fluctuations), we regressed each participant's 4-D pre-processed volume on nine predictors that modeled nuisance signals from white matter, cerebrospinal fluid, the global signal, and six motion parameters. Each participant's resultant 4-D residuals volume was spatially normalized by applying the previously computed transformation to MNI152 standard space, with 2 mm^3^ resolution.

#### Seed regions of interest

**Figure 4:**
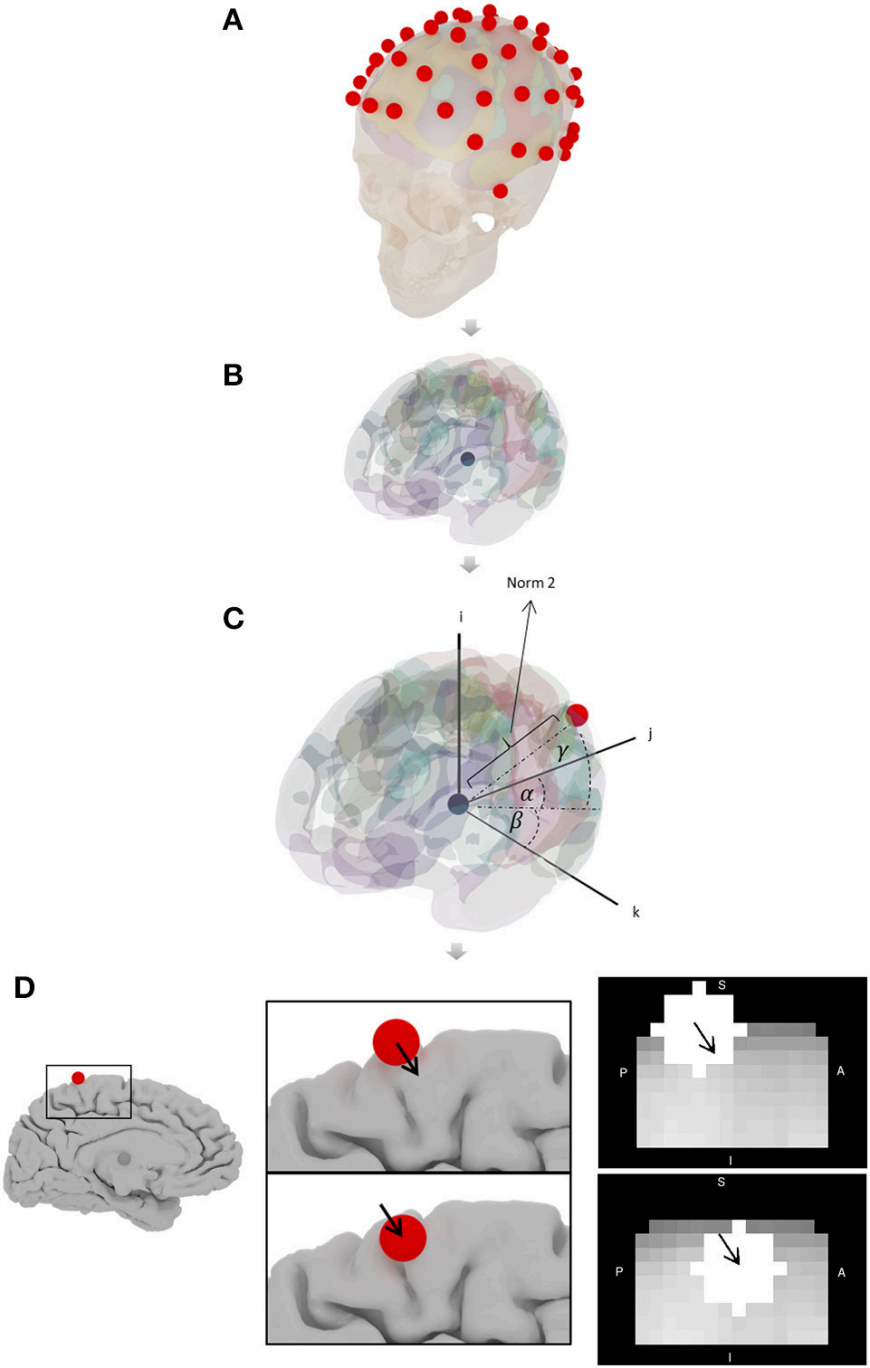
Algorithm to determine 10-10 EEG seeds coordinates. **(A)** Spheres in the head using Koessler coordinates (Oostenveld and Praamstra, 2001; Koessler et al., 2009), **(B)** center of mass of the brain, **(C)** magnitude and direction of the vector connecting the center of mass and the position of each electrode, and **(D)** magnitude of the vector is modified until the sphere is within the brain.

MNI coordinates corresponding to the 65 electrodes of the 10-10 EEG system were obtained. The algorithm to determine the coordinates related to 10-10 EEG seeds is:

- Position spheres on the head using MNI coordinates published by Koessler (Oostenveld and Praamstra, 2001; Koessler et al., 2009; Figure 4A).
- Determine the center of mass of the brain using 3DsMax software (Autodesk Inc., www.autodesk.com) and mesh created with MNI152_T1_2mm_brain_mask_nii.gz (fMRIB Software Library FSL; http://fsl.fmrib.ox.ac.uk/fsl/fslwiki; Smith et al., 2004; Jenkinson et al., 2012 Figure 4B).
- Calculate the magnitude and direction of the vector connecting the center of mass of the brain and the position of each electrode (Figure 4C).
- The magnitude of the vector is modified until the electrode sphere (4 mm radius) is completely within the brain by keeping α, β, γ angles unmodified (Figure 4D).

**Figure 5:**
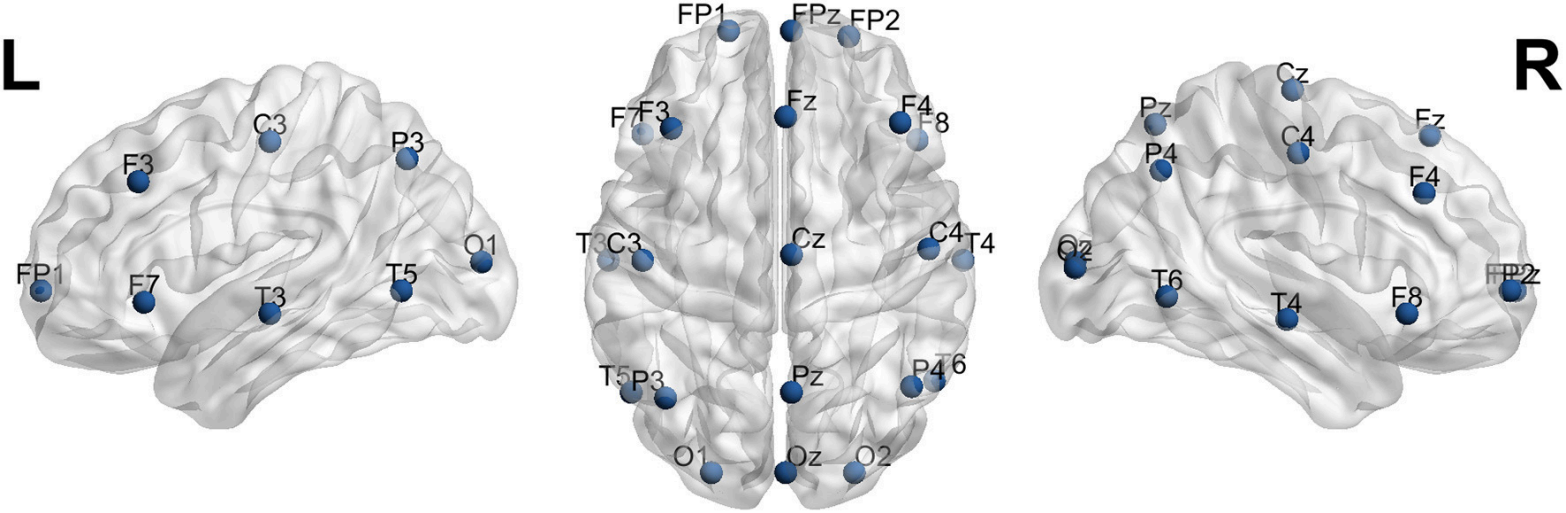
21 spherical ROIs over a brain surface (10-20 EEG electrode system). Figure created using BrainNetViewer software (http://www.nitrc.org/projects/bnv; Xia et al., 2013).

**Figure 6:**
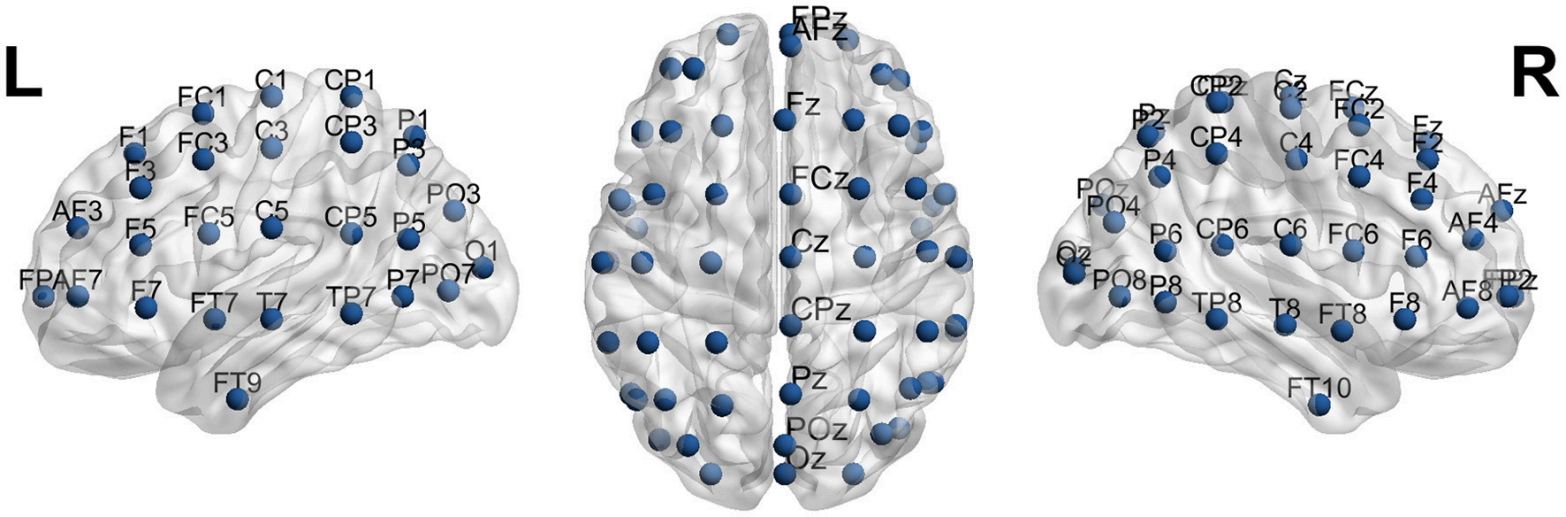
65 spherical ROIs over a brain surface (10-10 EEG electrode system). Figure created using BrainNetViewer software (http://www.nitrc.org/projects/bnv; Xia et al., 2013).

In Supplementary Table 1, the coordinates, brain lobes, hemisphere, brain region, Brodmann area and EEG electrode name of each 10-10 EEG ROIs are included. In Supplementary Table 2, the same data is included, but for the ROIs equivalent to the 10-20 EEG electrode system. In Figure 5, the 21 spherical ROIs are shown (10-20 EEG electrode system), and in Figure 6, the 65 spherical ROIs are shown over a brain surface (10-10 EEG electrode system). Figures 5, 6 were created using BrainNetViewer software (http://www.nitrc.org/projects/bnv; Xia et al., 2013).

#### Subject-level resting state functional connectivity (RSFC) analysis

For each participant, the representative time series for each 10-10 EEG (seed) ROI (see Supplementary Table 1) was extracted from their 4D residuals volume in standard space by averaging the time-series across all voxels within the ROI. We then calculated the Pearson's correlation coefficient between each seed ROI time series using a standard MATLAB®-based code. The resultant participant-level correlation maps were transformed via Fisher-z to Z-value maps and transformed into MNI152 2 mm standard space for group-level analyses.

A similar analysis such as the previous one has been carried out using the representative time series for each 10-20 EEG (seed) ROI.

#### Group-level RSFC analysis

For each seed, group-level analyses were carried out using a random-effects ordinary least squares model. Whole-brain correction for multiple comparisons was performed using Gaussian Random Field Theory (min Z>2.3; cluster significance: *p* < 0.05, corrected). This group-level analysis produced threshold z-score maps of activity associated with each 10-10 EEG and 10-20 EEG seed.

#### Functional connectivity networks comparison

To compare the similarity of functional connectivity mapping obtained using each 10-20 EEG electrode seeds and relative to the seven Yeo networks (Yeo et al., 2011), we compute the Sørensen-Dice similarity coefficient (Dice, 1945; Sørensen, 1948). Sørensen-Dice coefficient (also known as Dice index, Sørensen index, or F1 score) is a statistical index commonly used for comparing the similarity of two different samples and their outputs are in the interval between 0 and 1. The Sørensen coefficient was computed using the equation:

(1) $$\begin{array}{r} QS=\frac{2|X\cap Y|}{\left|X|+|Y\right|}, \end{array}$$

where |*X*|, and |*Y*| is the cardinality of sets X and Y respectively. In our case, |*X*| is the quantity of voxels of the functional connectivity networks obtained with each 10-20 EEG related seeds, and |*Y*| corresponds to the quantity of voxels of each of the seven Yeo networks (Yeo et al., 2011; https://surfer.nmr.mgh.harvard.edu/fswiki/CorticalParcellation_Yeo2011). In both cases we used MNI152 2 mm normalized functional connectivity networks.

## Results

**Figure 7:**
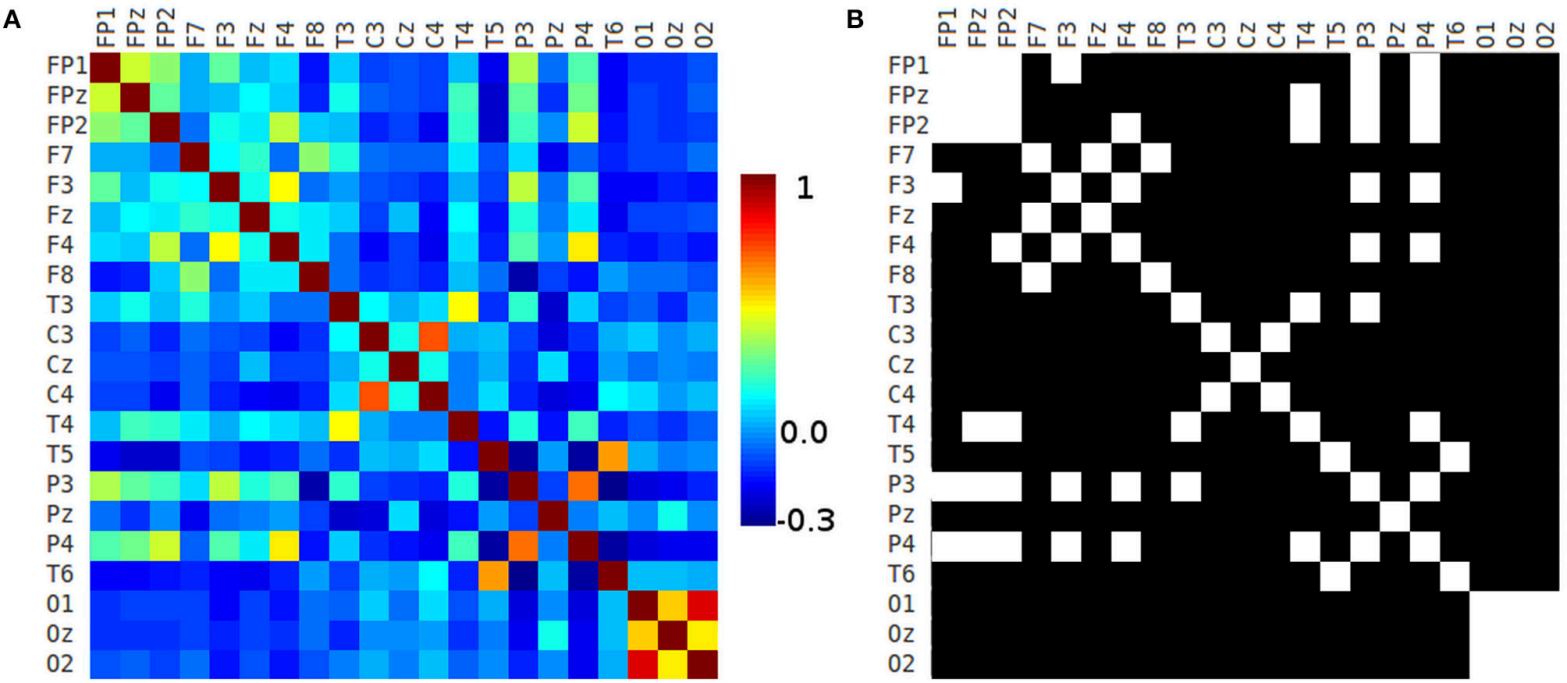
10-20 correlation matrix**. (A)** Correlation matrix of each 10-20 EEG electrode seed and **(B)** adjacency matrix generated using a threshold of 0.2.

We computed the correlation matrix of each 10-20 EEG electrode seed for each healthy volunteer. The mean matrix of the 45 correlation matrices of each healthy volunteer is depicted in Figure 7A.

The adjacency matrix is a square matrix which is used as a way of representing binary relations, and it shows more clearly the pairs of seeds that have correlation coefficient values greater than a given threshold. Figure 7A shows the Pearson's correlation matrix of the 10-20 EEG electrode system seeds. That matrix was analyzed using GRETNA software (Wang et al., 2015) computing the small-world sigma (Watts and Strogatz, 1998) using different thresholds (from 0.05 to 0.5 in 0.05 intervals) obtaining small-world sigma greater than 1.0 in all thresholds classifying the networks as small-world (Watts and Strogatz, 1998; Telesford et al., 2011). Figure 7B shows the adjacency matrix created by applying a threshold of 0.2 to the correlation matrix. There is strong connectivity between frontopolar and some frontal 10-20 EEG seeds. In Figures 7A,B appear contralateral strong connectivity between frontal, central, temporal, parietal, and occipital seeds. Also, P3 and P4 have strong connectivity with frontopolar and some frontal seeds. Occipital seeds have high connectivity between them.

**Figure 8:**
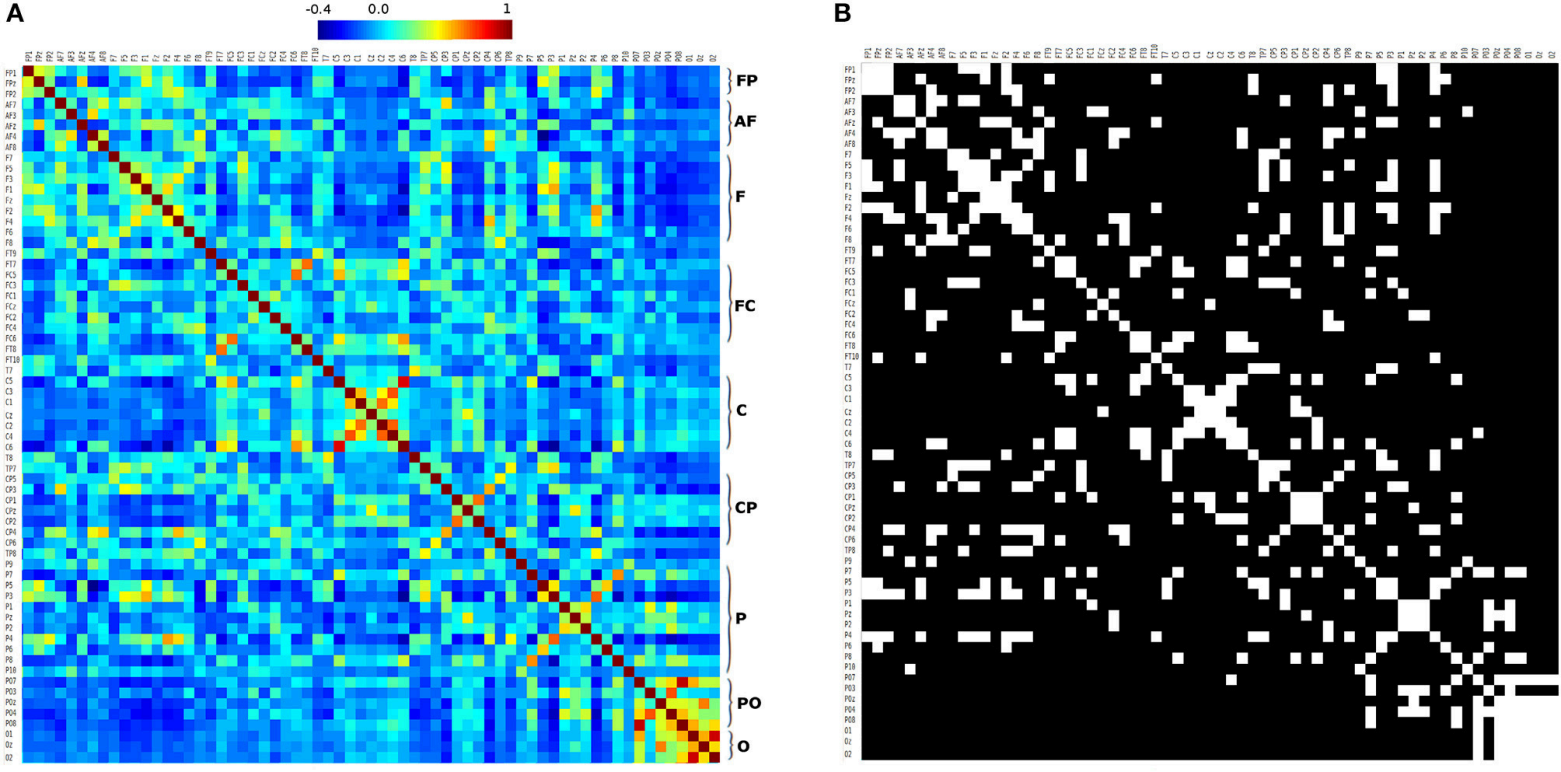
10-10 correlation matrix. **(A)** correlation matrix of 10-10 EEG electrode seed system, and **(B)** adjacency matrix generated using a threshold of 0.2. FP, Fronto-polar; AF, Anterior-frontal; F, Frontal; FC, Fronto-central; C, Central; CP, Centro-parietal; P, Parietal; PO, Parieto-occipital; O, Occipital.

Figure 8A shows the correlation matrix of the 10-10 EEG electrode system seeds. That matrix was analyzed using GRETNA software with same parameters used with 10-20 EEG electrode seeds correlation matrix. We obtained the small-world sigma greater than 1.0 in all cases classifying the networks as small-world (Watts and Strogatz, 1998; Telesford et al., 2011), and Figure 8B shows the adjacency matrix with a threshold of 0.2 (mean value of positive values of the correlation matrix). These results are similar to Figure 7, but with higher resolution (due to the fact that 10-10 EEG has more seeds than 10-20 EEG). Frontopolar, anterior-frontal, frontal, parieto-occipital, and occipital seeds have ipsilateral and contralateral strong connectivity. Some frontocentral, central, centroparietal and parietal seeds have contralateral high connectivity (Figures 8A,B).

**Figure 9:**
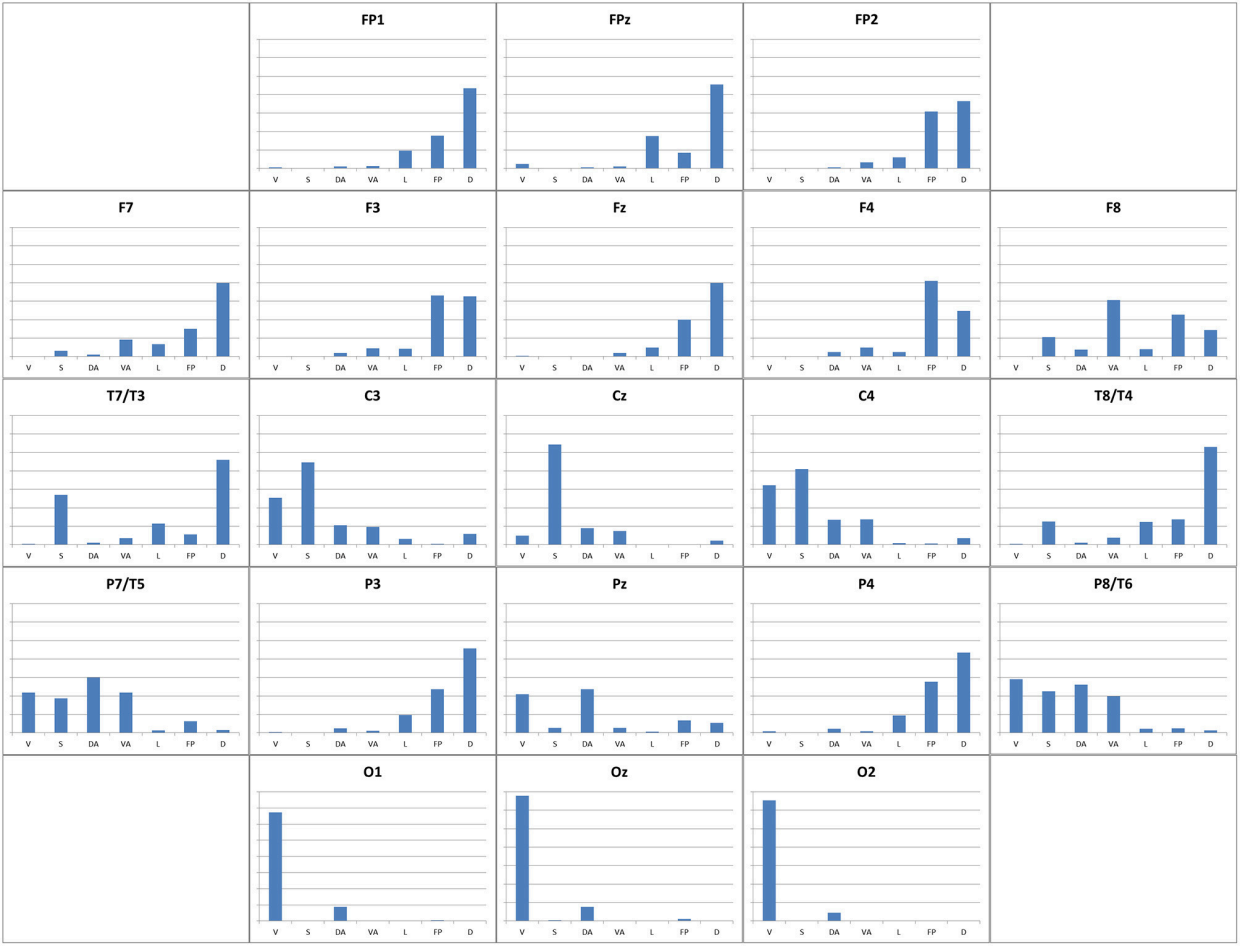
Similarity of 10-20 EEG seeds relative to Yeo networks. The similarity of functional connectivity mapping obtained using each 10-20 EEG seeds relative to seven Yeo networks (Yeo et al., 2011). The figure shows a bar chart for each EEG seed with Sørensen-Dice similarity coefficient (Dice, 1945; Sørensen, 1948) (see Materials and Methods section). In each bar chart X-axis shows Yeo networks (V, Visual; S, Somatomotor; DA, Dorsal attention; VA, Ventral attention; L, Limbic; FP, Frontoparietal; D, Default mode network; columns from left to right), and Y-axis shows Sørensen-Dice similarity coefficient (0.0–0.8 values).

We computed Sørensen-Dice coefficient. In Supplementary Table 3, we show the Dice similarity index related to Visual, Somatomotor, Dorsal Attention, Ventral Attention, Limbic, Frontoparietal, and Default mode networks. Figure 9 depicts the Dice indices as a bar chart in each 10-20 EEG seed. Broadly, Dice index does not have large values (less than 0.68), and higher-value Sørensen-Dice coefficients are in occipital seeds. Sørensen-Dice coefficient shows laterality for some Yeo networks.

**Figure 10:**
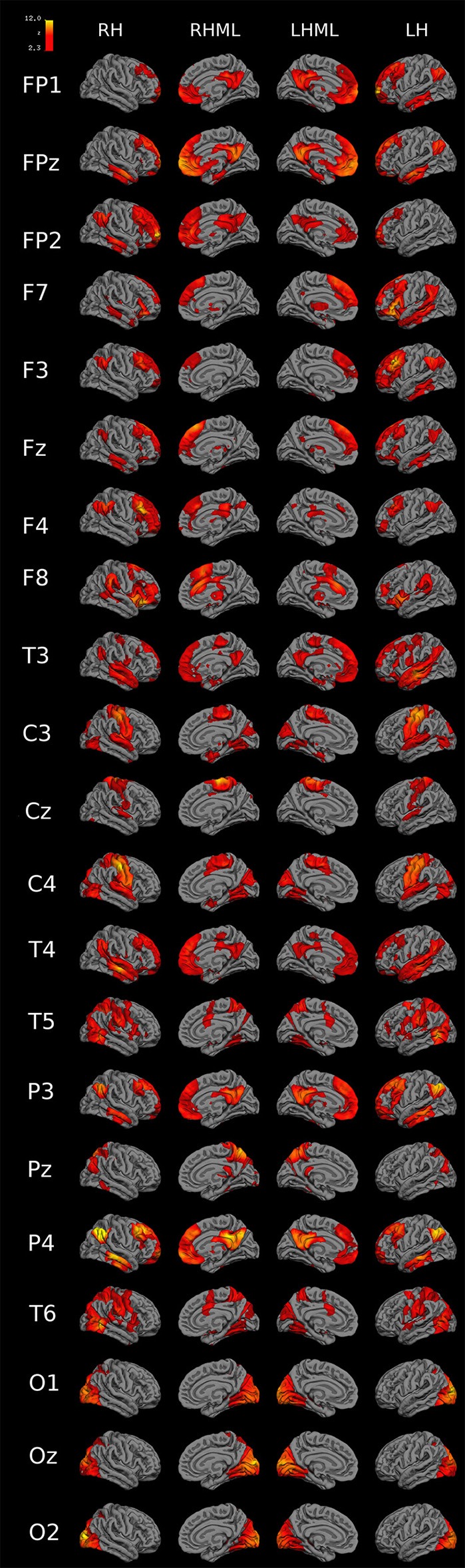
3D functional connectivity surface maps (FP1-O2 seeds). 3D connectivity images for each 10-20 EEG electrode-seed (FP1 to O2) created with 45 rs-fMRI images previously mentioned in Materials and Methods section (Cambridge-Buckner dataset, 1000 Functional Connectomes Project). Meaning of abbreviations: RH (right hemisphere), RHML (right hemisphere medial longitudinal fissure), LH (left hemisphere), LHML (left hemisphere medial longitudinal fissure).

Figure 10 shows 3D functional connectivity surface maps for each 10-20 EEG electrode seed. The maps were created with the 45 previously mentioned rs-fMRI images (Cambridge-Buckner dataset, 1000 Functional Connectomes Project).

In Figure 10 FP1, FP2 seeds showed connectivity with bilateral medial prefrontal cortex (MPF), bilateral posterior cingulate cortex (PCC), ipsilateral lateral parietal cortex (LP), and ipsilateral inferior temporal cortex (IT). FPz connects with bilateral MPF, bilateral PCC, left hemisphere LP, bilateral IT and bilateral parahippocampal cortex. F7 connects with bilateral thalamus, ventrolateral prefrontal cortex (VLPFC), IT, and MPF, and ipsilateral LP. F8 has connectivity with bilateral inferior parietal gyrus, anterior dorsolateral PFC (aDLPFC), ipsilateral MPF, contralateral dorsal anterior cingulate cortex (dACC), and insula. F3 connects with bilateral MPF, and LP, ipsilateral aDLPFC, and IT. F4 connects with ipsilateral aDLPFC, PCC, and bilateral LP. Fz has functional connectivity with bilateral MPF, IT, and LP. T3 and T4 have connectivity with bilateral LP, PCC, IT, middle temporal gyrus, and superior temporal gyrus, ipsilateral VLPFC and precentral gyrus (only with T3). C3 and C4 connect with bilateral pre/post central gyrus (PCG), posterior occipital cortex (pOCC), cuneus, superior temporal gyrus, parahippocampal gyrus, and fusiform gyrus. Cz has connectivity with bilateral PCG and superior temporal gyrus. T5 and T6 have connectivity with bilateral pOCC, IPS, PCG, superior frontal gyrus, superior temporal gyrus, lateral occipital-temporal gyrus, and aDLPFC (only in T6). P3 and P4 connect to bilateral LP, MPF, IT, middle temporal gyrus. Pz connects with right hemisphere LP, bilateral intraparietal sulcus (IPS), and PCC. O1 connect with bilateral precuneus and hippocampus, and ipsilateral cingulate gyrus. O2, with bilateral precuneus, hippocampus, and cingulate gyrus. Oz has a connection with bilateral precuneus and hippocampus and left hemisphere cingulate gyrus.

### Analysis for each functional connectivity network

In eleven EEG seeds (FP1, FP2, FPz, F3, F4, F7, Fz, T3, T4, P3, P4), the Sørensen-Dice coefficient (Figure 9) of the **Default Mode Network** is greater than 0.2, and the left hemisphere EEG seeds have a higher Sørensen-Dice coefficient than those of the right side (left lateralized functional connectivity network). Our results are consistents with (Agcaoglu et al., 2015) that specify the same lateralization of the Default mode network for young people, and similar left lateralization conclusions reports (Nielsen et al., 2013) and (Swanson et al., 2011) for healthy people.

The **Frontoparietal Network** is right-lateralized because it has a higher percentage of functional connectivity voxels in even numbered EEG seeds than in odd numbered seeds. The Frontoparietal Network coexists with the default mode network (more than 35% of maximum value Sørensen-Dice index; 0.143 and 0.186 respectively) in multiple frontal seeds (Fp1, Fp2; BA 10, F3, F4; BA 9, FZ; BA 8), anterior temporals (F7, BA 47) and parietal seeds (P3, P4, BA 39).

**Ventral Attention Network:** the Sørensen-Dice coefficient has the highest value in F8 seed (BA 47; 0.306). Other high values of Sørensen-Dice coefficient are in T5, T6, C4 (0.218, 0.199, and 0.138 respectively).

C3 (BA 3), C4 (BA 3), T5 (BA 19), T6 (BA 19), and Pz (BA 7), have a Dice index greater than 0.105 (35% of maximum value of Dice index) for the **Dorsal Attention Network**. This network is not lateralized (4.3% difference between left and right mean Sørensen-Dice coefficient).

**Limbic Functional Network** is also left-lateralized because in general, odd numbered EEG seeds have higher Dice index than the even numbered ones. And because, the percent difference between left and right mean Sørensen-Dice coefficient is more than 20%.

The **Somatomotor functional network** has the highest Sørensen-Dice index in Cz, (BA 6), C3 (BA 3) and C4 (BA 3) EEG seeds (0.544, 0.447, and 0.410 respectively). In general, we could conclude that it is an equally lateralized network (less than 8% difference between both hemispheres of Sørensen-Dice coefficient).

The **Visual Functional Network** has a Dice index greater than 0.600 in O1 (BA 19; Associative visual cortex V3, V4, V5), 02 (BA 18; secondary visual cortex V2), and Oz seeds. In C4 (BA 3), T6 (BA 19), and C3 (BA 3) the Visual Network has a Sørensen-Dice coefficient more than 0.25. In the connectivity images for O1, O2, and Oz appears to have connectivity only with occipital and parietal regions (Figures 8, 10, Supplementary Table 3). Using Dice index (10% difference) we could specify that Visual functional network is right lateralized as specified (Agcaoglu et al., 2015).

## Discussion

Epilepsy and other neurological diseases are diagnosed with EEG. In this paper, we show rs-fMRI results and analysis processed in a similar way as EEG.

In most clinical cases, the 10-20 EEG electrodes scheme is used adding some 10-10 EEG electrodes, and, therefore, functional connectivity was processed in a similar way as EEG, i.e., using 10-20 and 10-10 EEG related seeds.

We described a new method to study brain connectivity using EEG electrode position as seed and then we use the seeds to get, study and analyze rs-fMRI based functional connectivity and different brain networks.

Our main interest is to study in a future work the connectivity in comparison to EEG electrode influence, besides to detect with the source estimation method how to get the real seizure onset zone in epileptic networks (Martinez-Vargas et al., 2017).

On the basis of the correlation and 10-20 adjacency matrixes (Figures 7A,B), it can be suggested the following:

Broadly, there is a high correlation between contralateral seeds (for instance FP1-FP2, F3-F4, F7-F8, C3-C4, T3-T4, P3-P4, and T5-T6). Also, there is a high correlation between ipsilateral seeds (FP2-F4, FP1-F3, FP2-T4), high correlation between frontopolar, frontal and anterior temporal seeds with parietal seeds (FP1-P3, FP2-P3, FPz-P3, F3-P3, F3-P4, F4-P3, F4-P4 and T3-P3), and high correlation between occipital seeds: O1-O2, Oz-O1, Oz-O2.

The 10-10 correlation matrix (Figure 8A), suggests that:

There is a high connectivity between frontal, frontopolar, anterior-frontal, and frontocentral EEG seeds, between ipsilateral and contralateral seeds (adjacency matrix with a threshold of 0.2 in Figure 8B). There is a high contralateral connectivity between centroparietal, and parietal EEG seeds.

There is high connectivity between the occipital EEG seeds (between ipsilateral and contralateral seeds; Figure 8B).

The occipital EEG seeds (O1, O2, Oz) only have connectivity with PO7 (Figure 8B).

### General analysis

The fact that more than one network coexists in a single seed (see bar charts in Figure 9) can be explained because the brain is and works as a network of interconnections (or graph-connected) where the existing networks are not independent and must be connected to function as an integrated organ. If we assume that networks are independent, different functional areas such as memory, visual or, motor could not be inter-related, contradicting the fact that the brain works as a network of interconnections.

Seeds located according to 10-20 EEG system in frontopolar, frontal, anterior temporal and parietal regions show high regional connectivity in relation to the default mode and frontoparietal networks. The default mode network predominates in the left hemisphere, probably this being related to a higher representativity of this functional network to the left (Agcaoglu et al., 2015), whereas in the right hemisphere the frontoparietal networks predominate (Agcaoglu et al., 2015). The frontoparietal network, located between the default mode network and the dorsal attention network, has a role in goal-directed cognition (Vincent et al., 2008; Spreng et al., 2013), and in the integration of information from the dorsal attention network and the default mode network (Vincent et al., 2008).

Also, frontal contralateral seeds have high connectivity (Figures 7, 8, 10). Similar results were published in an ICA-derived EEG functional connectivity study that shows the power spectra of each independent component (Brodmann area 10, Figure 1C in Chen et al., 2013), and other EEG connectivity based paper that specify that contralateral frontal connections are common (61%, Figure 3 in Lacruz et al., 2007).

Central level seeds (C3, C4, and Cz) have high connectivity between each other with significant somatomotor representation (primary motor-sensory area; Figures 10, 11), and moderate connectivity with temporal posterior region seeds (Figures 10, 11) coinciding with the dorsal attention network (functional area related to processing of external sensory stimuli), ventral attention network, and not connected with the default mode network region (Buckner et al., 2013).

Seeds in the occipital region (visual area) have high ipsilateral and contralateral connectivity slightly connected to some dorsal attention network regions (precuneus, cingulate gyrus, and hippocampus Figure 10). The primary sensory areas (visual, somatomotor) and the limbic area would be evolutionarily older than other parts of the prosencephalon, which is consistent with a modular-type organization, probably determined phylogenetically. Primary sensory areas participate in simple networks with local networks preferably. The greater the local connectivity is, the less functional variability exists, i.e., the occipital region is a stable connectivity area between the individuals (robust networks) and, therefore, would have no variability with respect to their connections. Instead, areas represented by seeds in associative area regions, mainly prefrontal, temporal and parietal, coinciding with frontoparietal and attention networks, have connectivity above the global mean of the inter-subject variability (Yeo et al., 2013). Fahoum et al. (2012) shows that in posterior quadrant epilepsy there exist only deactivation clusters in bilateral PCC and precuneus, not activations.

Associative areas have high long-range connections that are related to greater functional variability. Apparently, distant connectivity is necessary for prominent functional variability, and this is not observed in species with smaller brains. On the other hand, association areas are more recent in the evolution of the human brain development, whose greater functional variability could also be explained by its later maturation during development, which would make these more vulnerable to external postnatal influences and less dependent on genetic factors (Mueller et al., 2013).

Being able to establish normal connectivity models through combination (or fusion) of methods -in this case, electric signals and hemodynamic data- allows us to address the temporal and spatial relationship of the observed connections, and, in the future, to correlate it with connectivity in epilepsy patients (or other brain disease), in order to establish abnormal association networks so as to not only try to refine the detection of seizure origin area using non-invasive methods, but also to predict surgical prognosis, response to drug therapy, or cognitive impairment, inter allia.

The functional connectivity analysis of the rs-fMRI using 10-20 EEG positioned seeds as proposed in this article makes it possible to obtain connectivity maps superimposed with networks described by Yeo et al. (2013) and Brodmann areas Brodmann (1909), determining comparable connectivity matrices.

Functional connectivity analysis of rs-fMRI using 10-10 or 10-20 EEG positioned seeds has the following benefits: helps the medical doctor to understand the connectivity alterations detected by fMRI using classical parameters of EEG localization (standard EEG electrode position). EEG is currently the standard method for monitoring or diagnosis of some diseases (Schomer and Lopes da Silva, 2011; Ebersole et al., 2014). Also, using the proposed scheme it is possible to perform a correlation analysis between the anatomical layout of EEG surface electrodes and Yeo functional connectivity networks (see iBraiNEEG mobile device application described in Rojas et al., 2016; and freely available for Android at https://goo.gl/zFrWNP; iOS from https://goo.gl/9vULy9; Yeo et al., 2011). This analysis scheme will helps to analyze RS-fMRI data acquired simultaneously with EEG. In a future work, we pretend compare the results obtained with the EEG data analysis, with the resting state data analyzed with the scheme proposed in this work.

Finally, the objective of our study was to provide a replicable model in which the arrangement of seeds using 10-20 EEG system allows us to combine methods for temporal and spatial location through noninvasive markers, in order to identify abnormal functional networks.

## Author contributions

GR: article writing, creation of figures, design of paper, design of methods, processing and analysis of data. CA: article writing, analysis of data. CM: article writing, creation of figures, design of methods. MdlI-V: creation of figures, article review and correction. JC: article writing, article review and correction. MG: article review and correction, analysis of data.

### Conflict of interest statement

The authors declare that the research was conducted in the absence of any commercial or financial relationships that could be construed as a potential conflict of interest.
