# Supplementary material for: Study of Resting-State Functional Connectivity Networks Using EEG Electrodes Position As Seed
Source: Front Neurosci. 2018 Apr 24;12:235. doi: 10.3389/fnins.2018.00235 (PMC5928390; doi:10.3389/fnins.2018.00235)
Supplement: Supplementary file 1 [file Table_1.docx]

Supplementary Material

**Study of Resting-State Functional Connectivity Networks using EEG electrodes position as seed.**

Gonzalo M. Rojas^*^, Carolina Alvarez, Carlos Montoya, María de la Iglesia-Vayá, Jaime Cisternas, Marcelo Gálvez

*** Correspondence:** Corresponding Author: gonzalo.rojas.costa@gmail.com

# Supplementary Data

# Supplementary Figures and Tables

## Supplementary Tables

| **Seed** | **MNI Coordinates** | | | **Lobe (Hemisphere)** | **Region** | **BA^a^** |
| --- | --- | --- | --- | --- | --- | --- |
|  | **X** | **Y** | **Z** |  |  |  |
| FP1 | -18 | 62 | 0 | Frontal Lobe (L) | Medial Frontal Gyrus | 10 |
| FPz | 4 | 62 | 0 | Frontal Lobe (R) | Medial Frontal Gyrus | 10 |
| FP2 | 24 | 60 | 0 | Frontal Lobe (R) | Superior Frontal Gyrus | 10 |
| AF7 | -38 | 50 | 0 | Frontal Lobe (L) | Middle Frontal Gyrus | 10 |
| AF3 | -30 | 50 | 24 | Frontal Lobe (L) | Superior Frontal Gyrus | 9 |
| AFz | 4 | 58 | 30 | Frontal Lobe (R) | Medial Frontal Gyrus | 9 |
| AF4 | 36 | 48 | 20 | Frontal Lobe (R) | Middle Frontal Gyrus | 10 |
| AF8 | 42 | 46 | -4 | Frontal Lobe (R) | Inferior Frontal Gyrus | 10 |
| F7 | -48 | 26 | -4 | Frontal Lobe (L) | Inferior Frontal Gyrus | 47 |
| F5 | -48 | 28 | 18 | Frontal Lobe (L) | Inferior Frontal Gyrus | 45 |
| F3 | -38 | 28 | 38 | Frontal Lobe (L) | Precentral Gyrus | 9 |
| F1 | -20 | 30 | 50 | Frontal Lobe (L) | Superior Frontal Gyrus | 8 |
| Fz | 2 | 32 | 54 | Frontal Lobe (L) | Superior Frontal Gyrus | 8 |
| F2 | 26 | 32 | 48 | Frontal Lobe (R) | Superior Frontal Gyrus | 8 |
| F4 | 42 | 30 | 34 | Frontal Lobe (R) | Precentral Gyrus | 9 |
| F6 | 50 | 28 | 14 | Frontal Lobe (R) | Middle Frontal Gyrus | 46 |
| F8 | 48 | 24 | -8 | Frontal Lobe (R) | Inferior Frontal Gyrus | 47 |
| FT9 | -50 | -6 | -36 | Temporal Lobe (L) | Inferior Temporal Gyrus | 20 |
| FT7 | -54 | 2 | -8 | Temporal Lobe (L) | Superior Temporal Gyrus | 22 |
| FC5 | -56 | 4 | 22 | Frontal Lobe (L) | Precentral Gyrus | 6 |
| FC3 | -44 | 6 | 48 | Frontal Lobe (L) | Middle Frontal Gyrus | 6 |
| FC1 | -22 | 6 | 64 | Frontal Lobe (L) | Middle Frontal Gyrus | 6 |
| FCz | 4 | 6 | 66 | Frontal Lobe (R) | Medial Frontal Gyrus | 6 |
| FC2 | 28 | 8 | 60 | Frontal Lobe (R) | Sub-Gyral | 6 |
| FC4 | 48 | 8 | 42 | Frontal Lobe (R) | Middle Frontal Gyrus | 6 |
| FC6 | 58 | 6 | 16 | Frontal Lobe (R) | Inferior Frontal Gyrus | 44 |
| FT8 | 54 | 2 | -12 | Temporal Lobe (R) | Superior Temporal Gyrus | 38 |
| FT10 | 50 | -6 | -38 | Temporal Lobe (R) | Inferior Temporal Gyrus | 20 |
| T7/T3 | -60 | -18 | -8 | Temporal Lobe (L) | Middle Temporal Gyrus | 21 |
| C5 | -62 | -18 | 24 | Parietal Lobe (L) | Postcentral Gyrus | 2 |
| C3 | -48 | -18 | 52 | Parietal Lobe (L) | Postcentral Gyrus | 3 |
| C1 | -24 | -18 | 70 | Frontal Lobe (L) | Precentral Gyrus | 4 |
| Cz | 4 | -16 | 70 | Frontal Lobe (R) | Medial Frontal Gyrus | 6 |
| C2 | 30 | -16 | 66 | Frontal Lobe (R) | Precentral Gyrus | 4 |
| C4 | 52 | -14 | 48 | Parietal Lobe (R) | Postcentral Gyrus | 3 |
| C6 | 62 | -16 | 18 | Parietal Lobe (R) | Postcentral Gyrus | 43 |
| T8/T4 | 64 | -18 | -10 | Temporal Lobe (R) | Middle Temporal Gyrus | 21 |
| TP7 | -60 | -46 | -6 | Temporal Lobe (L) | Middle Temporal Gyrus | 37 |
| CP5 | -60 | -46 | 22 | Parietal Lobe (L) | Inferior Parietal Lobule | 40 |
| CP3 | -46 | -46 | 54 | Parietal Lobe (L) | Inferior Parietal Lobule | 40 |
| CP1 | -22 | -46 | 70 | Parietal Lobe (L) | Postcentral Gyrus | 7 |
| CPz | 4 | -40 | 68 | Frontal Lobe (R) | Paracentral Lobule | 5 |
| CP2 | 30 | -42 | 68 | Parietal Lobe (R) | Postcentral Gyrus | 5 |
| CP4 | 52 | -42 | 50 | Parietal Lobe (R) | Inferior Parietal Lobule | 40 |
| CP6 | 62 | -40 | 18 | Temporal Lobe (R) | Superior Temporal Gyrus | 13 |
| TP8 | 62 | -42 | -8 | Temporal Lobe (R) | Middle Temporal Gyrus | 20 |
| P9 | -46 | -52 | -40 | Posterior Lobe (L) | Cerebellar Tonsil | Cerebellum |
| P7/T5 | -52 | -64 | 0 | Temporal Lobe (L) | Inferior Temporal Gyrus | 19 |
| P5 | -50 | -66 | 20 | Occipital Lobe (L) | Middle Temporal Gyrus | 19 |
| P3 | -40 | -66 | 46 | Parietal Lobe (L) | Inferior Parietal Lobule | 39 |
| P1 | -20 | -68 | 56 | Parietal Lobe (L) | Precuneus | 7 |
| Pz | 4 | -64 | 58 | Parietal Lobe (R) | Precuneus | 7 |
| P2 | 28 | -66 | 56 | Parietal Lobe (R) | Precuneus | 7 |
| P4 | 46 | -62 | 42 | Parietal Lobe (R) | Angular Gyrus | 39 |
| P6 | 52 | -60 | 16 | Occipital Lobe (R) | Middle Temporal Gyrus | 19 |
| P8/T6 | 54 | -60 | -2 | Temporal Lobe (R) | Inferior Temporal Gyrus | 19 |
| P10 | 46 | -52 | -38 | Posterior Lobe (R) | Cerebellar Tonsil | Cerebellum |
| PO7 | -42 | -80 | 2 | Occipital Lobe (L) | Inferior Occipital Gyrus | 19 |
| PO3 | -32 | -82 | 30 | Occipital Lobe (L) | Superior Occipital Gyrus | 19 |
| POz | 2 | -82 | 32 | Occipital Lobe (L) | Cuneus | 18 |
| PO4 | 36 | -78 | 26 | Occipital Lobe (R) | Middle Occipital Gyrus | 19 |
| PO8 | 42 | -76 | 0 | Occipital Lobe (R) | Inferior Occipital Gyrus | 19 |
| O1 | -24 | -92 | 10 | Occipital Lobe (L) | Middle Occipital Gyrus | 19 |
| Oz | 2 | -92 | 10 | Occipital Lobe (L) | Lingual Gyrus | 17 |
| O2 | 26 | -92 | 8 | Occipital Lobe (R) | Middle Occipital Gyrus | 18 |

^a^ **BA**: Brodmann area

**Supplementary Table 1.** **MNI Coordinates of 10-10 EEG related seeds.**
