# Supplementary material for: Study of Resting-State Functional Connectivity Networks Using EEG Electrodes Position As Seed
Source: Front Neurosci. 2018 Apr 24;12:235. doi: 10.3389/fnins.2018.00235 (PMC5928390; doi:10.3389/fnins.2018.00235)
Supplement: Supplementary file 2 [file Table_2.docx]

Supplementary Material

**Study of Resting-State Functional Connectivity Networks using EEG electrodes position as seed.**

Gonzalo M. Rojas^*^, Carolina Alvarez, Carlos Montoya, María de la Iglesia-Vayá, Jaime Cisternas, Marcelo Gálvez

*** Correspondence:** Corresponding Author: gonzalo.rojas.costa@gmail.com

# Supplementary Data

# Supplementary Figures and Tables

## Supplementary Tables

| **Seed** | **MNI Coordinates** | | | **Lobe (Hemisphere)** | **Region** | **BA^a^** |
| --- | --- | --- | --- | --- | --- | --- |
|  | **X** | **Y** | **Z** |  |  |  |
| FP1 | -18 | 62 | 0 | Frontal Lobe (L) | Medial Frontal Gyrus | 10 |
| FPz | 4 | 62 | 0 | Frontal Lobe (R) | Medial Frontal Gyrus | 10 |
| FP2 | 24 | 60 | 0 | Frontal Lobe (R) | Superior Frontal Gyrus | 10 |
| F7 | -48 | 26 | -4 | Frontal Lobe (L) | Inferior Frontal Gyrus | 47 |
| F3 | -38 | 28 | 38 | Frontal Lobe (L) | Precentral Gyrus | 9 |
| Fz | 2 | 32 | 54 | Frontal Lobe (L) | Superior Frontal Gyrus | 8 |
| F4 | 42 | 30 | 34 | Frontal Lobe (R) | Precentral Gyrus | 9 |
| F8 | 48 | 24 | -8 | Frontal Lobe (R) | Inferior Frontal Gyrus | 47 |
| T7/T3 | -60 | -18 | -8 | Temporal Lobe (L) | Middle Temporal Gyrus | 21 |
| C3 | -48 | -18 | 52 | Parietal Lobe (L) | Postcentral Gyrus | 3 |
| Cz | 4 | -16 | 70 | Frontal Lobe (R) | Medial Frontal Gyrus | 6 |
| C4 | 52 | -14 | 48 | Parietal Lobe (R) | Postcentral Gyrus | 3 |
| T8/T4 | 64 | -18 | -10 | Temporal Lobe (R) | Middle Temporal Gyrus | 21 |
| P7/T5 | -52 | -64 | 0 | Temporal Lobe (L) | Inferior Temporal Gyrus | 19 |
| P3 | -40 | -66 | 46 | Parietal Lobe (L) | Inferior Parietal Lobule | 39 |
| Pz | 4 | -64 | 58 | Parietal Lobe (R) | Precuneus | 7 |
| P4 | 46 | -62 | 42 | Parietal Lobe (R) | Angular Gyrus | 39 |
| P8/T6 | 54 | -60 | -2 | Temporal Lobe (R) | Inferior Temporal Gyrus | 19 |
| O1 | -24 | -92 | 10 | Occipital Lobe (L) | Middle Occipital Gyrus | 19 |
| Oz | 2 | -92 | 10 | Occipital Lobe (L) | Lingual Gyrus | 17 |
| O2 | 26 | -92 | 8 | Occipital Lobe (R) | Middle Occipital Gyrus | 18 |

^a^ **BA**: Brodmann area

**Supplementary Table 2**. **MNI Coordinates of 10-20 EEG related seeds.**
