# Supplementary material for: Study of Resting-State Functional Connectivity Networks Using EEG Electrodes Position As Seed
Source: Front Neurosci. 2018 Apr 24;12:235. doi: 10.3389/fnins.2018.00235 (PMC5928390; doi:10.3389/fnins.2018.00235)
Supplement: Supplementary file 3 [file Table_3.docx]

Supplementary Material

**Study of Resting-State Functional Connectivity Networks using EEG electrodes position as seed.**

Gonzalo M. Rojas^*^, Carolina Alvarez, Carlos Montoya, María de la Iglesia-Vayá, Jaime Cisternas, Marcelo Gálvez

*** Correspondence:** Corresponding Author: gonzalo.rojas.costa@gmail.com

# Supplementary Data

# Supplementary Figures and Tables

## Supplementary Tables

|  | **Visual** | **Somatomotor** | **Dorsal Attention** | **Ventral Attention** | **Limbic** | **Frontoparietal** | **Default** |
| --- | --- | --- | --- | --- | --- | --- | --- |
| **FP1** | 0,0069 | 0,0000 | 0,0116 | 0,0138 | 0,0965 | 0,1778 | 0,4355 |
| **FP2** | 0,0012 | 0,0003 | 0,0054 | 0,0336 | 0,0597 | 0,3079 | 0,3653 |
| **FPz** | 0,0238 | 0,0002 | 0,0065 | 0,0108 | 0,1757 | 0,0854 | 0,4545 |
| **F7** | 0,0000 | 0,0320 | 0,0109 | 0,0920 | 0,0677 | 0,1506 | 0,3989 |
| **F8** | 0,0000 | 0,1047 | 0,0371 | 0,3058 | 0,0413 | 0,2269 | 0,1430 |
| **F3** | 0,0000 | 0,0000 | 0,0193 | 0,0444 | 0,0416 | 0,3305 | 0,3260 |
| **F4** | 0,0000 | 0,0000 | 0,0254 | 0,0482 | 0,0248 | 0,4098 | 0,2484 |
| **Fz** | 0,0030 | 0,0000 | 0,0012 | 0,0209 | 0,0502 | 0,1995 | 0,3985 |
| **T7/T3** | 0,0048 | 0,2695 | 0,0101 | 0,0367 | 0,1136 | 0,0554 | 0,4587 |
| **T8/T4** | 0,0032 | 0,1256 | 0,0115 | 0,0389 | 0,1247 | 0,1365 | 0,5303 |
| **C3** | 0,2543 | 0,4466 | 0,1046 | 0,0971 | 0,0309 | 0,0040 | 0,0575 |
| **C4** | 0,3221 | 0,4102 | 0,1345 | 0,1379 | 0,0089 | 0,0063 | 0,0353 |
| **Cz** | 0,0499 | 0,5435 | 0,0895 | 0,0729 | 0,0026 | 0,0027 | 0,0227 |
| **P7/T5** | 0,2178 | 0,1878 | 0,2993 | 0,2181 | 0,0141 | 0,0620 | 0,0143 |
| **P8/T6** | 0,2916 | 0,2242 | 0,2617 | 0,1986 | 0,0218 | 0,0250 | 0,0137 |
| **P3** | 0,0031 | 0,0001 | 0,0233 | 0,0102 | 0,0959 | 0,2368 | 0,4569 |
| **P4** | 0,0081 | 0,0001 | 0,0224 | 0,0088 | 0,0936 | 0,2768 | 0,4352 |
| **Pz** | 0,2088 | 0,0262 | 0,2368 | 0,0272 | 0,0068 | 0,0670 | 0,0548 |
| **O1** | 0,6718 | 0,0007 | 0,0869 | 0,0007 | 0,0010 | 0,0048 | 0,0002 |
| **O2** | 0,6547 | 0,0000 | 0,0442 | 0,0000 | 0,0015 | 0,0029 | 0,0000 |
| **Oz** | 0,6785 | 0,0039 | 0,0763 | 0,0004 | 0,0000 | 0,0104 | 0,0010 |

**Supplementary Table 3**. **Sørensen-Dice coefficient**. Sørensen-Dice coefficient (or Dice index; Sørensen, 1948; Dice, 1945) of functional connectivity mapping (obtained using each 10-20 EEG electrode seeds) relative to seven Yeo networks (Yeo et al., 2011). See Material and methods section and Figure 9.
